# Case Report: Laparoscopic vaginoplasty in a case of partial androgen insensitivity syndrome and a literature review of 16 cases in China

**DOI:** 10.3389/fsurg.2025.1702539

**Published:** 2025-12-10

**Authors:** Haixia Qin, Huaqian Liu, Qiang Liu, Baiou Li

**Affiliations:** 1 Beijing IKang Bayley & Jackson Medical Center, Beijing, China; 2 Beizhen Prison Hospital, Liaoning Province, China; 3 Minimally Invasive & Reproductive Medicine Centre, The Affiliated Hospital of Northwest University, Angel Obstetrics and Gynecology Hospital, Xi’an, China

**Keywords:** androgen insensitivity syndrome, urogenital abnormalities, female masculinization, laparoscopy, vaginoplasty, acellular dermal matrix

## Abstract

**Background:**

We examined the clinical features, diagnosis, treatment, and future gender choice of patients with partial androgen insensitivity syndrome (PAIS).

**Case presentation:**

The clinical features, specialty examinations, three-stage surgical examination, and treatment process of a patient with PAIS were comprehensively reviewed. Additionally, 16 PAIS case reports were collected from Chinese databases and analyzed. Seventeen PAIS cases were included in this study. All patients presented with primary amenorrhea (17/17) and displayed specific clinical features such as a male social identity or appearance (3/17), an underdeveloped phallus or clitoromegaly (16/17), abnormal urethral development (7/17), and breast development (4/17). Twelve cases were managed as women, both surgically and with hormone replacement therapy. The patient in this case report underwent three surgical procedures involving laparoscopic examination and clitoroplasty, left orchidopexy, and laparoscopic vaginoplasty using peritoneum and an acellular dermal matrix.

**Conclusions:**

PAIS is a rare disorder of sex development (DSD) with a 46,XY karyotype and is a congenital X-linked recessive disease. Therefore, a deeper understanding of the pathogenesis of androgen insensitivity syndrome allows more accurate diagnosis, personalized treatment, and organized follow-up of the condition, thereby avoiding gender dysphoria.

## Introduction

Androgen insensitivity syndrome (AIS) is a disorder of sex development (DSD) with a 46, XY karyotype and is classified as either complete AIS (CAIS) or partial AIS (PAIS). Since PAIS is less common than CAIS and is associated with virilization, treatment plans are determined based on the severity of external genital malformation and findings from surgical examination. Three patients with special characteristics of PAIS have been treated at our hospital. One patient was diagnosed at the age of 15 in 2015, and the treatment plan was confirmed after the initial laparoscopic examination. The patient subsequently underwent an open orchiectomy and estrogen replacement therapy at the age of 17, followed by a laparoscopic vaginoplasty at the age of 24, completing the entire treatment plan. A literature review of databases in China revealed several cases of PAIS, which are summarized and retrospectively analyzed below.

## Case presentation

### Patient

**Figure 1:**
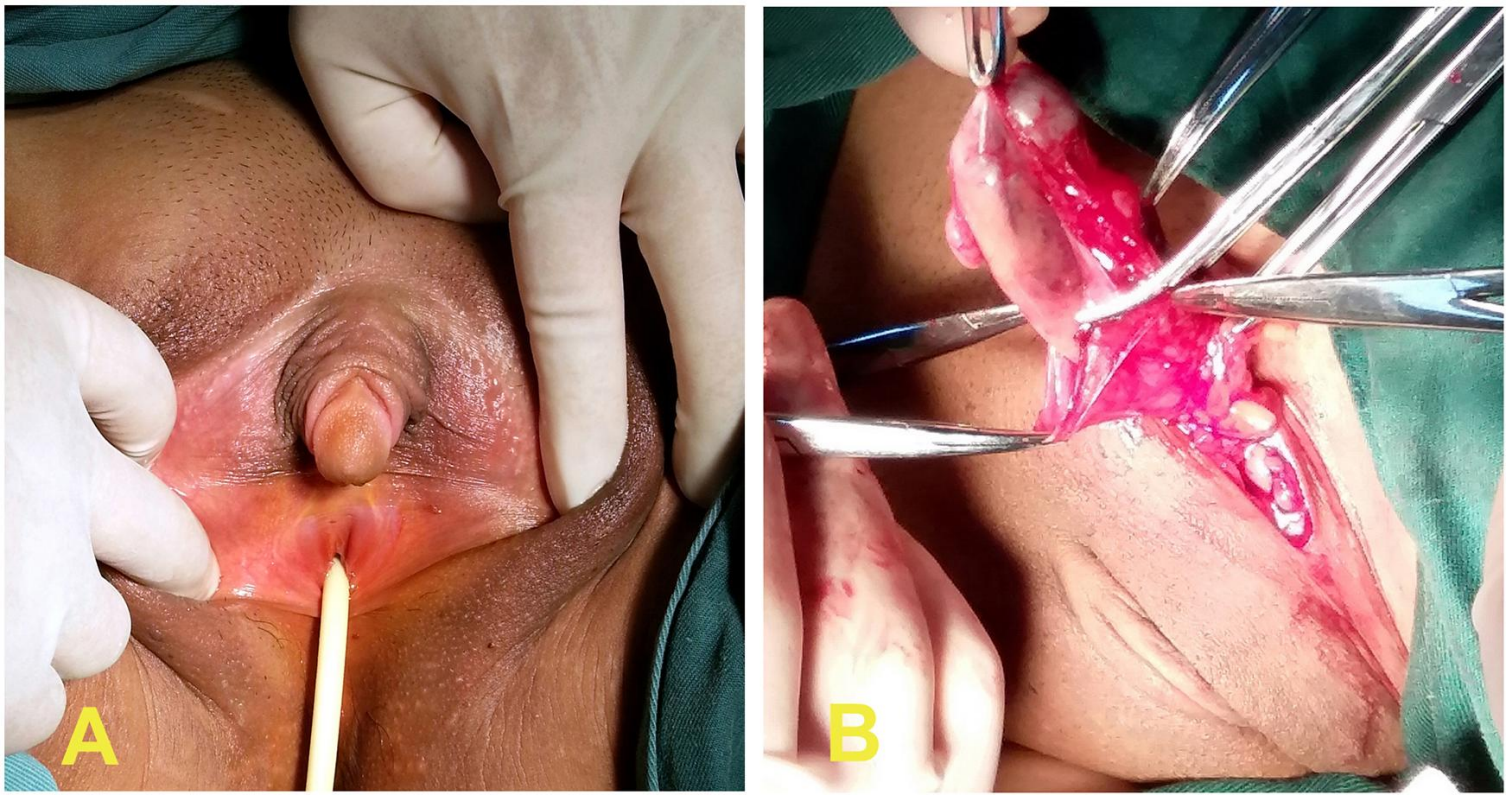
**(A)** A microphallus measuring approximately 3.0 cm × 2.0 cm was observed, with a coronary sulcus but no urethral opening. The urethral opening was located at the posterior commissure of the vestibule, just above the normal vaginal opening. **(B)** A cord-like structure measuring approximately 5.0 × 2.0 cm in the left inguinal region was observed, representing the testicle.

A 15-year-old student, socially identified as a woman, was admitted to our hospital due to atypical external genital development that had been apparent for one month. One month prior, the patient was initially suspected of having “pancreatitis” and was found to have atypical external genitalia during a physical examination at a local hospital. The patient had a 46, XY karyotype and was subsequently referred to our hospital for further diagnosis. The patient had no history of menstruation or periodic abdominal pain. Her family reported a history of “hernia” (right inguinal) repair 10 years prior, but the specifics were unknown. Her mother denied any history of exposure to harmful toxins or drugs during pregnancy. Physical examination: Female appearance, a height of 172 cm, a weight of 76 kg, slight laryngeal prominence, a hoarse voice, a strong physique, breast development at Tanner stage Ⅰ, pale areolas, small nipples, and no axillary hair. A 3 cm scar was visible to the right of the mons pubis, and a peanut-sized mobile nodule was palpated deep within the left labia majora. Specialty examination: Dense pubic hair with a female distribution pattern. A microphallus measuring approximately 3.0 cm × 2.0 cm was observed, with a coronary sulcus but no urethral opening. The urethral opening was located at the posterior commissure of the vestibule, just above the vaginal-like opening (Figure 1A). No vagina nor uterus were identified during a rectal examination. An abdominal ultrasound revealed an absence of a uterus or bilateral adnexa in the pelvic cavity. The kidneys, ureters, and bladder appeared normal. Serum hormones: FSH: 43.72 IU/L, LH: 19.64 IU/L, E2: 40.63 pmol/L, P: 0.92 nmol/L, T: 3.12 nmol/L, PRL: 430.36 mIU/L. Admission diagnosis: PAIS. (The reference ranges for male patients are as follows: Follicle-Stimulating Hormone (FSH): 1.5–12.4 IU/L; Luteinizing Hormone (LH): 1.7–8.6 IU/L; Estradiol (E2): 27.9–156.3 pmol/L; Progesterone (P): 0.6–4.5 nmol/L; Testosterone (T): 9.7–27.8 nmol/L; Prolactin (PRL): 84.8–322.2 mIU/L).

### Diagnosis and treatment process

Laparoscopic examination revealed an empty pelvic cavity without a uterus, fallopian tubes, or ovaries. The gonads were not found along the pelvic peritoneum up to the deep inguinal ring. Penile reduction and vulvoplasty were performed, and the pathological diagnosis of the excised clitoral tissue revealed: a stratified squamous epithelium with spongy tissue visible in the deep layer of the subepithelial stroma, confirming the penile tissue and consistent with the preoperative diagnosis. Therefore, it is recommended for these patients to use a vaginal mold or engage in sexual activity, if appropriate for their age, to avoid stenosis or occlusion after surgery, the family requested that vaginoplasty and other surgeries be performed after the age of 18. However, they were advised to return for follow-up after three months and informed of the risks associated with not removing the gonads. One year later, the patient underwent pelvic and pituitary MRI scans, which revealed: 1. Absence of a uterus or bilateral adnexa; 2. An oval-shaped atypical signal around the urethra below the bladder, suggesting a prostate. Oval-shaped atypical signals on either side of the posterior region of the bladder base, suggesting seminal vesicles; 3. Atypical signals in the left inguinal region, suggesting cryptorchidism; 4. A “bulging” pituitary gland without a significant atypical signal. The patient was readmitted in 2017. An inguinal ultrasound revealed a hypoechoic area measuring 4.3 cm × 2.8 cm in the lower left inguinal region with distinct boundaries. On the same day, the inguinal mass (approximately 5 cm × 2.0 cm) was successfully removed under epidural anesthesia. The cord-like mass was oval on one end, resembling a testicle (Figure 1B). Pathology results indicated the presence of testicular, epididymal, and spermatic cord tissues (left inguinal region). A 6-month telephone follow-up revealed no complications.

**Figure 2:**
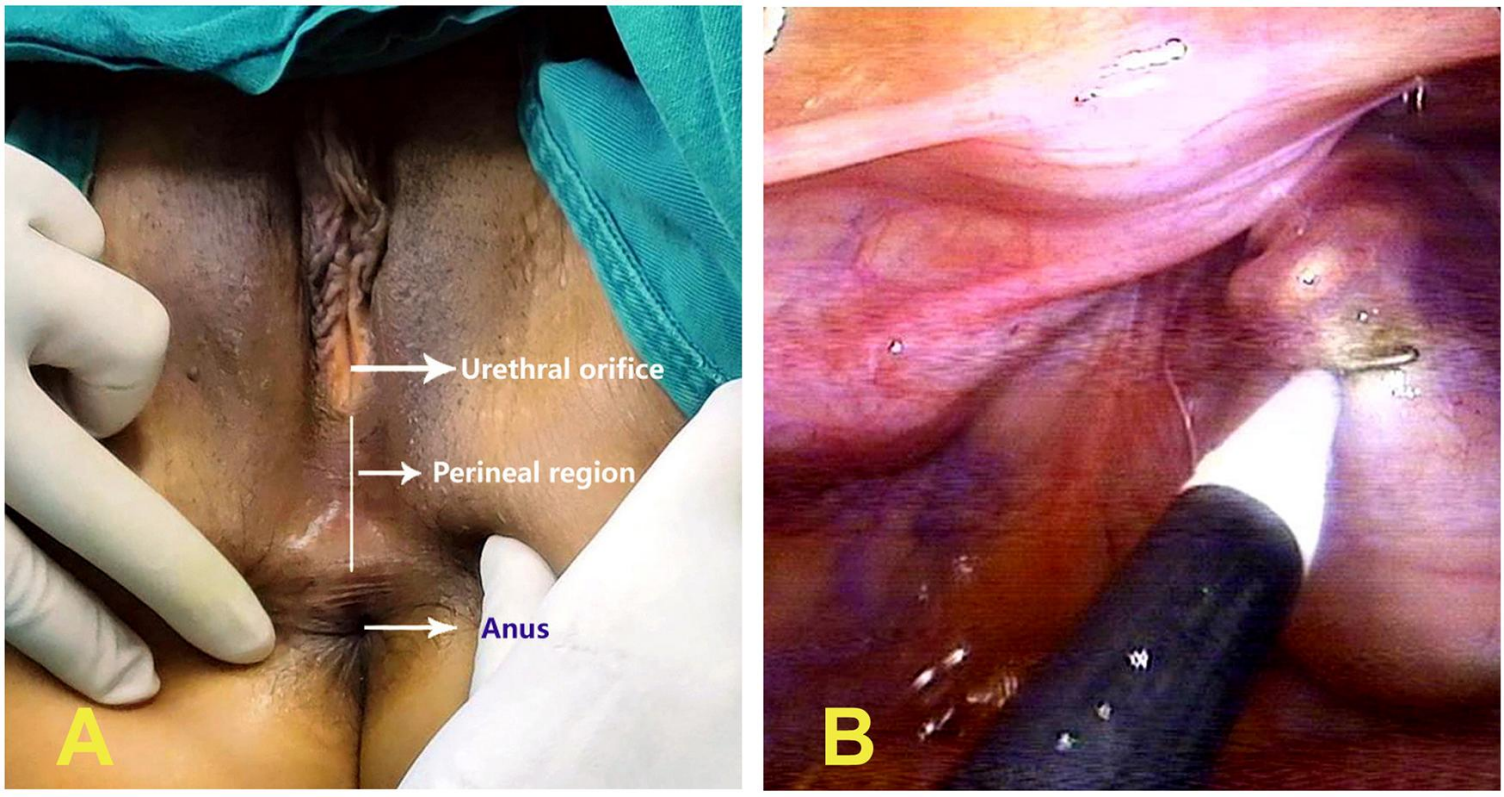
**(A)** absence of vestibular mucosa between the normal urethral opening and the anus, with the perineum covered by skin. **(B)** Incision of the most prominent area by electrocautery.

**Figure 3:**
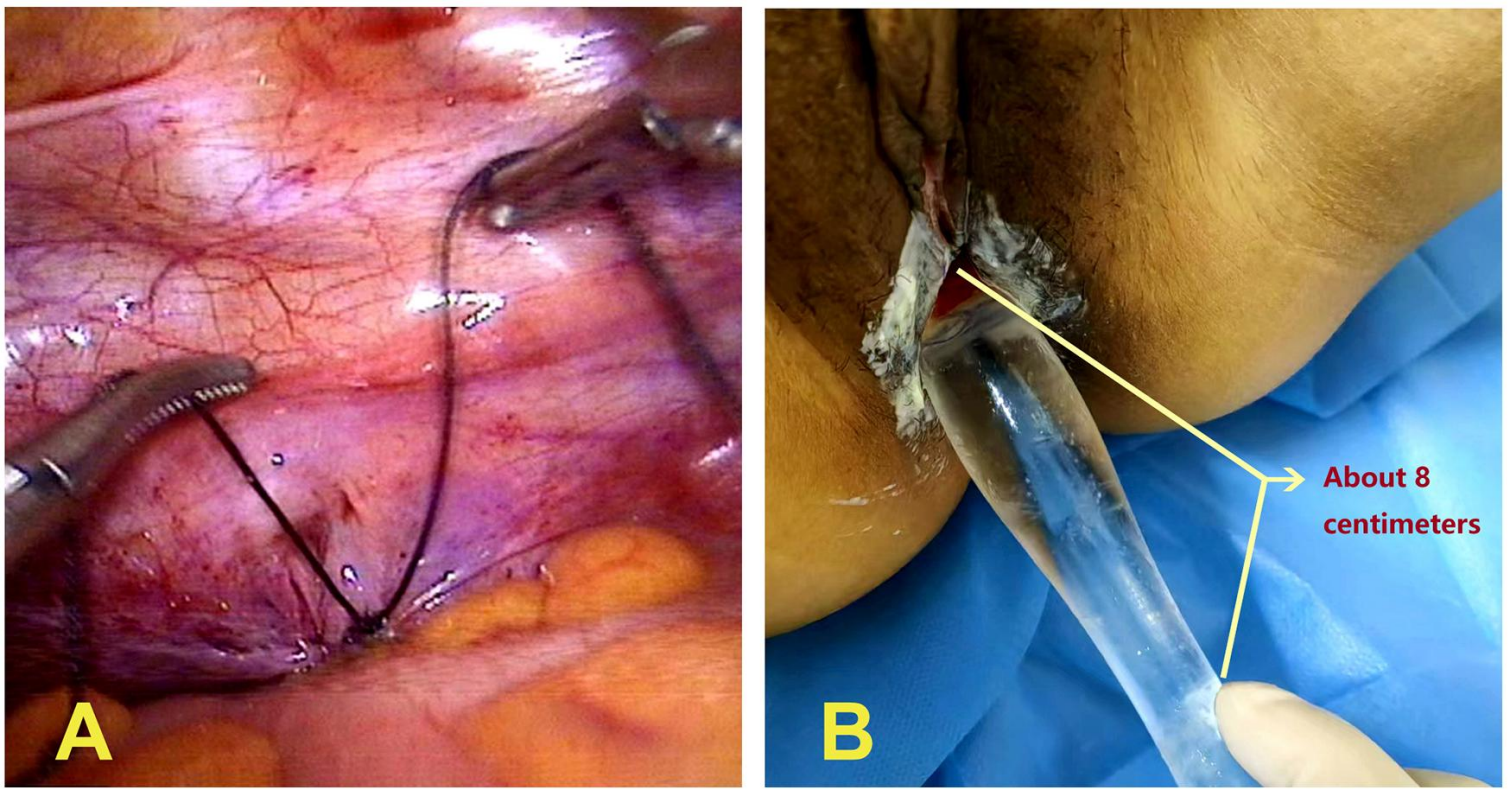
**(A)** closure of the pelvic floor peritoneum at the top of the neovagina by purse-string suture using #7 non-absorbable sutures. **(B)** Twelve days after surgery, the depth of the neovagina was about 8 cm deep, and a vaginal mold could be inserted and removed smoothly.

In 2024, the patient was admitted for the third time. After penile excision, vulvoplasty, orchiectomy, and estrogen replacement therapy (Specific plan: 5 mg of diethylstilbestrol three times a day and taken continuously for one year, with function liver checked once every three months), the microphallus was no longer visible during specialized examinations, and the patient's breasts progressed to stage V development and appeared similar to those of a typical woman of the same age. The patient was scheduled for vaginoplasty; however, due to her 46,XY DSD, which differs from Mayer-Rokitansky-Küster-Hauser syndrome, there was no vestibular mucosa between the normal urethral opening and the anus. Instead, the perineum was covered with skin (Figure 2A). Four days later, laparoscopic peritoneal vaginoplasty was performed. A cavity was created in the skin along the midline between the urethral opening and the anus, separating and extending toward the pelvic cavity until reaching the pelvic floor peritoneum. The most prominent area was incised using an electrocautery (Figure 2B) to enlarge and expand the artificial cavity to accommodate two fingers. Under laparoscopy, the edge of the pelvic floor peritoneum was grasped with Maryland forceps, and the neovagina was pulled outward from the abdominal cavity. The surgical assistant used Allis forceps to grasp the pelvic floor peritoneum from the outer opening of the neovagina and pull it inward until reaching two-thirds of the neovagina, and fixed it with an absorbable suture. Because the pelvic peritoneum was not long enough to reach the external opening of the neovagina, one-third of the neovagina wound was covered with an acellular dermal matrix (ADM), while the remaining portion was sutured at multiple sites. The pelvic floor peritoneum at the apex of the neovagina was closed with a purse-string suture using #7 non-absorbable sutures (Figure 3A). The surgery lasted 72 min and was completed successfully. Twelve days after surgery, the depth of the neovagina was about 8 cm, allowing for easy insertion and removal of the vaginal mold (Figure 3B). The patient was discharged 15 days after the surgery. Follow-up at six months showed that the neovagina was approximately 9 cm in depth, and that the ADM had integrated well with the surgical wound. There was a trace amount of discharge, but no contact bleeding. The patient was instructed to continue neovagina dilation to prevent contracture and stenosis. Since stenosis or occlusion of the artificial vagina may occur due to peritoneal and ADM coverage, in October 2025, we asked the patient to return to the hospital for a follow-up visit. Although the artificial vagina was slightly shallower than at the time of surgery, we used a smooth glass mold with a diameter of 2.5 cm. After coating the surface with a lubricant, it could enter the artificial vagina relatively smoothly, with a depth of approximately 7 cm. The patient did not experience any obvious discomfort or pain. Therefore, it is necessary for such patients to use a mold and engage in sexual activity after surgery.

### Literature review of 16 cases in China

We searched the China National Knowledge Infrastructure (CNKI), Wanfang Database, Chinese Biomedical Literature Database (CBM), and Chinese Clinical Case Results Database (CMCR) using the keywords “incomplete/partial androgen insensitivity syndrome”. A total of 16 case reports were retrieved, involving 16 patients ranging in age from 2 months to 29 years. The cases are ranked by publication year and summarized along with the clinical data of the present case in Table 1. Large-scale clinical analysis articles were not included due to the potential for duplication. Moreover, this article only summarizes the cases found in China.

**Table 1:** Clinical data of 16 patients with PAIS in China.

Serial no./Age (years)	Presentations/symptoms	Height (cm)	Secondary sexual characteristics	Specialty examination/Karyotype	Treatment process/pathology/sex selection
1/27	External genital abnormality/Primary amenorrhea	165	Female appearance, well-developed muscles, delicate skin, no mustache, unremarkable laryngeal prominence, low and dull voice. Tanner stage I-II breast development	Male pattern of pubic hair distribution, underdeveloped phallus (approximately 4.5 cm), and erection after stimulation. Prominent bilateral labia majora, but no vagina or labia minora. Non-palpable uterus or ovaries on rectal examination/46, XY	Penile corpus cavernosum excision/Underdeveloped testicular tissues in intra-abdominal gonads/female (21)
2/28	Primary amenorrhea	155	Socially identified as a woman/girl, no laryngeal prominence, no mustache, sparse axillary hair, low and dull voice. No breast development	Female-like external genitalia, no scrotum, urethral opening in the perineum, vaginal opening posterior to the urethral opening. Phallus was approximately 3.5 cm long, with painful erections. Non-palpable uterus or adnexa on rectal examination/46, XY	Perineal hypospadias, bilateral inguinal cryptorchidism, bilateral inguinal hernia/male (22)
3/25	External genital abnormality/Primary amenorrhea	162	Socially identified as a woman/girl, laryngeal prominence, coarse voice, no mustache, sparse axillary hair. No breast development	Female-like external genitalia and underdeveloped penile-like tissues (approximately 3 cm) that bend toward the abdomen, with painful erections. No scrotum and no vaginal opening visible. No uterus or bilateral adnexa/46, XY with large Y (15)	Congenital hypospadias, left inguinal and right intra-abdominal cryptorchidism/female (23)
4/20	External genital abnormality/Primary amenorrhea	168	Male appearance, well-developed muscles, coarse skin, no mustache, prominent laryngeal prominence, no breast development	Female-like external genitalia with pubic hair in an inverted triangle shape, enlarged clitoris (approximately 2.0 cm × 1.0 cm) with painful erections, vaginal opening was visible but could not accommodate one finger (probe depth of approximately 3 cm). Rectal examination: Empty pelvis, with non-palpable uterus, bilateral adnexa or pelvic mass/(46, XY) at the 320–400 band level	Laparoscopic bilateral cryptorchidectomy/underdeveloped bilateral testicular tissues, consistent with cryptorchidism/female (24)
5/29	External genital abnormality/Primary amenorrhea	166	Female appearance, no laryngeal prominence, delicate skin, high-pitched voice, sparse body hair, no mustache, well-developed breasts	Gynecological examination: Dense pubic hair in an inverted triangle shape. Mixed external genitalia. Enlarged phallus and glans (approximately 3.0 cm × 1.0 cm) with painful erection, loose urethral opening, mucosal eversion, and no vagina. Rectal examination: non-palpable uterus or bilateral adnexa/46, XY	Laparoscopic ileal vaginoplasty + bilateral orchiectomy + partial clitoridectomy/bilateral testicular and epididymal tissues/female (25)
6/15	Bilateral breast enlargement for 3 years	160	Socially identified as a man/boy, no laryngeal prominence, no mustache or pubic/axillary hair. Bilateral breast development (Tanner stage II)	Hypospadias, phallus approximately 2.5 cm long, bilateral testes with a volume of approximately 6 mL, soft texture/46, XY	Open orchidopexy/male (26)
7/16	Primary amenorrhea	170	Laryngeal prominence, non-coarse voice, increased upper limb hair, sparse axillary hair, Tanner stage I breast development	Immature external genitalia, no pubic hair, vaginal atresia, 3 cm long vaginal pouch upon probing, non-palpable uterus or bilateral adnexa/46, XY	Laparoscopic bilateral inguinal cryptorchidectomy, epididymis excision/bilateral testicular seminiferous tubules without spermatogenic cells, underdeveloped epididymal tissue around the testis, absence of ovarian tissues/female (27)
8/7	Clitoromegaly for 3 years	119	Socially identified as a man/boy, no abnormal appearance, fair skin, bilateral stage B1 breasts, non-pigmented areolae	Female external genitalia, phallus of 2.0 cm × 0.8 cm, urethral and vaginal openings visible beneath the phallus/46, XY	Ultrasound revealed bilateral hypoechoic masses in the inguinal canal, suggesting testes. No surgery was performed/female (28)
9/23	External genital abnormality/Primary amenorrhea	178	Socially identified as a woman/girl, female appearance, sparse body hair, no mustache or laryngeal prominence, breast development consistent with that of age-matched women/girls	Clitoromegaly resembling a microphallus, commissure of labia majora, labia minora and vestibule, urethral opening at the dorsal root of the microphallus, vaginal opening not visible/46, XY	Bilateral mastectomy + orchidopexy + penile correction + stage I urethral repair/male (29)
10/14	External genital abnormality for 3 years	Not recorded	Socially identified as a woman/girl, perioral hair, sparse axillary hair, unremarkable laryngeal prominence, Tanner stage 0 breast development	Dense pubic hair in an inverted triangle pattern, short phallus-like structure (approximately 3 cm), urethral opening at the dorsal root of the microphallus, no scrotum or testes, no vaginal opening/46, XY	Cryptorchidectomy + clitoroplasty + axial flap transfer/female (29)
11/20	Inguinal mass present for 16 years	Not recorded	Female appearance, no laryngeal prominence	Immature external genitalia, enlarged phallus (approximately 3 cm), vaginal atresia (approximately 4 cm in depth), non-palpable uterus or adnexa, right inguinal mass approximately 4 cm × 2 cm with soft texture and well-defined boundaries, but no tenderness/46, XY	Laparoscopic bilateral cryptorchidectomy/Pathology results showed testicular tissues/Sigmoid colon vaginoplasty performed 1 year after surgery/female (30)
12/24	Hypospadias for 24 years, bilateral breast development for 9 years	160	Large nipples with female-like development. Sparse mustache, sparse axillary and body hair, unremarkable laryngeal prominence, gradual bilateral breast development	Short phallus with a length of approximately 5–6 cm after erection, small semen volume, pubic hair distributed in an inverted triangle pattern, Tanner stage IV; urethral opening below the glans of the phallus/46, XY (small Y)	Hypospadias repair/male (31)
13/17	Bilateral breast development for 2 years	154	Male appearance, breast development, Tanner stage IV mammary glands, no axillary hair, no other secondary sexual characteristics	Tanner stage III-IV male external genitalia, Tanner stage II pubic hair, no axillary hair/46, XY	Male sex selected, and mastectomy performed/male (32)
14/24	External genital abnormality/Primary amenorrhea	154	Undetermined phenotype, Tanner stage I breast development, non-pigmented areolae and nipples, nipples centrally located and approximately 6 mm in diameter/152 cm arm span	Enlarged phallus (approximately 3.5 cm × 1.5 cm) with a visible coronal sulcus. Retractable foreskin, plump bilateral labia majora, and no separate urethral opening, as the urethral and vaginal openings shared a common orifice. Rectal examination: Non-palpable uterus/46, XY (20)	Laparoscopic and open surgery/Pathology/female (33)
15/15	Amenorrhea	155	Indistinguishable phenotype, stage II breast development/height equal to arm span	Dense pubic hair, no visible vaginal vestibule or urethral opening, unremarkable labia minora. Elongated and enlarged phallus (4.0 cm × 2.0 cm). High posterior commissure, 9.0 cm deep vagina on probing (anterior to posterior commissure), visible urethral opening below the vaginal opening. Rectal examination revealed a non-palpable uterus or mass/46, XY (20)	Laparoscopy/Pathology/female (33)
16/2 months	External genital abnormality for 2 months and 22 days	0.59	Female at birth, external genital abnormalities observed after birth, manifested as thickened labia majora, enlarged phallus, and unclear urethral opening	Female-like external genitalia, thickened and loose labia majora resembling a scrotum, non-palpable mass, enlarged phallus (approximately 2.0 cm × 1.0 cm), urethra and vagina shared a common orifice, external genital virilization score: 2, Tanner stage I pubic hair/47, XXXY (whole-exome sequencing revealed a heterozygous variant in the AR gene: c.1847G>A, p.Arg616His)	Continued outpatient follow-up after multidisciplinary consultation with endocrinology, psychology, and urology, and parents; surgery plan will be determined before puberty/Not determined (18)

## Methods of analysis

The entire treatment plan and three surgical procedures for our PAIS case were described in detail. The clinical features, specialty examinations, gender orientation, treatment plans, and outcomes of the present case, along with the 16 cases identified in the literature, were tabulated.

## Results

All 17 patients were diagnosed through inquiry of medical history, clinical signs, specialty examinations, laboratory tests, and findings from laparoscopic and/or open surgery. In total, 10 of the patients initially presented as phenotypic women/girls, and all had primary amenorrhea. Case 16 was a recently reported case of Klinefelter syndrome (KS) with PAIS in an infant who was 2 months and 22 days old. Atypical external genitalia were observed at birth, presenting as clitoromegaly. Sex hormone testing was consistent with that of age-matched children, but karyotyping revealed 47, XXY. Due to severely insufficient virilization, whole-exome sequencing was performed on the child. The sequencing results indicated a heterozygous variant in the AR gene (c.1847G > A, p.Arg616His), confirming the diagnosis of PAIS. Of note, the child's older sister was diagnosed with CAIS. The infant is currently being followed up, and a multidisciplinary comprehensive evaluation is required to determine the treatment plan.

A combined analysis of the 16 case reports from the domestic literature and our current case revealed that 52.9% (9/17) of patients presented with “amenorrhea” as their chief complaint at the time of consultation. Although the remaining 8 cases primarily presented with breast development or clitoromegaly, amenorrhea was also present, indicating that primary amenorrhea is the most prominent clinical feature of PAIS. Of the 17 cases, 2 had an undetermined phenotype, 10 socially identified as women/girls or had a female appearance, and 5 socially identified as men/boys or had a male appearance. It is worth noting that only 3 of the 17 cases were ≥170 cm in height, and these cases socially identified as women/girls. In addition, among the 17 cases, 2 had no records, and 2 were under 7 years old. Specialty examinations revealed an underdeveloped phallus or clitoromegaly in 16 cases, with lengths ranging from 2.0 to 4.5 cm. Only 1 case did not include a description of this feature. Three cases also presented with congenital hypospadias, and two cases had urethral openings at the dorsal root of the microphallus. Four patients identifying as men in social contexts underwent bilateral mastectomy/ orchidopexy/penile correction/hypospadias repair (4/17). The remaining 12 patients received treatment to align their social identity with female characteristics. One patient, who was only 2 months old, did not receive further treatment. Gender-affirming treatments included open surgery, minimally invasive surgery, or hormone replacement therapy.

## Discussion

AIS, formerly known as testicular feminization, is a congenital X-linked recessive condition that causes DSD. It arises from complete or partial resistance to androgens in individuals with a 46, XY karyotype (1). Approximately 70% of AIS cases are due to X-linked recessive inheritance, while the remaining 30% occur in patients with no family history and may be attributed to AR gene mutations (2). Individuals with a 46,XY karyotype have normal male levels of testosterone and urinary 17-ketosteroids, along with testes. However, their testosterone cannot be converted into dihydrotestosterone due to a deficiency in 5-alpha reductase or they lack androgen expression due to the absence of dihydrotestosterone receptors, leading to the feminization of the external genitalia. Although the relationship between AIS phenotype and genotype has been increasingly investigated, the correlation between the two remains unclear (3). The clinical presentation of individuals with a 46, XY karyotype is that they appear as seemingly normal but infertile women. The testes of these individuals are typically retained within the abdominal cavity, with no spermatogenesis.

The incidence of AIS reported in the English literature is 1/20,000–99,000 female births. CAIS is the more common type of AIS, with a 10-fold higher incidence compared to PAIS (4). In addition to CAIS and PAIS, patients with AIS with normal male external genitalia have been further classified as mild AIS (MAIS) (5). The Cambridge University AIS patient database revealed that 95% of CAIS patients, but only 25% of PAIS patients, carry genomic aberrations (6). A study in South Korea found that 2/3 (6 out of 9) of CAIS patients and only 1/3 of PAIS patients had androgen receptor mutations (5), suggesting that PAIS is less common.

From a clinical perspective, the clinical manifestations of PAIS vary depending on the degree of androgen response in the external genitalia. PAIS can manifest clinically as nearly normal virilization to nearly complete feminization, or ambiguous external genitalia to a broad range of virilization processes, including mild hypospadias. Various phenotypes of PAIS include microphallus, severe hypospadias, gynecomastia, a bifid scrotum, cryptorchidism, and infertility. Additionally, PAIS may present with nearly normal female features, except for clitoromegaly and/or posterior labial fusion (5). In this study, over half (10/17) of the 17 PAIS patients presented with a female appearance or socially identified as women/girls, while 4 cases with significant breast development presented with a distinct phallus but no vagina. These 4 cases represented a significant discrepancy between the development of secondary sexual characteristics and the external genitalia, with a prominent phallus and developed breasts, but no vagina. Case 13 presented with a male appearance but developed breasts at the age of 15, reaching a Tanner stage IV within two years, which is very uncommon. Laparoscopic examination of case 14 revealed a small uterine remnant in the right pelvis, with a right fallopian tube connected to the uterine horn, but no visible gonads. The left pelvis was empty, and a testis was found in the left inguinal canal, which was highly unusual. Postoperative hormone replacement therapy resulted in significant changes in serum hormone levels. Since 5% of AIS patients may develop testicular malignancy after puberty, and PAIS has a higher malignancy rate of up to 30%, the risk is particularly elevated if the testes do not descend into the scrotum (7). Therefore, Kravarusic et al. (8) recommended that gonads should not be removed immediately upon diagnosis of PAIS to prevent malignancy. Furthermore, other studies have suggested that the risk of cancer increases with age, exceeding 30% in patients aged 50 and older (9). In our case, laparoscopic examination did not reveal any gonads; however, ultrasound and MRI identified a mass with atypical echoes/signals in the left inguinal region. This mass was excised and confirmed to be male gonadal tissue. Post-gonadectomy hormone replacement therapy and appropriate management and psychological counseling are crucial for helping these patients live normal lives.

Breast development is generally absent in young PAIS patients after puberty. Case 15 presented with Tanner stage II breast development, whereas our case and case 14 had no breast development at all. However, Lee et al. (10) reported a case of PAIS with gynecomastia during puberty (Tanner stage III). Aside from breast enlargement, there were no complaints of erectile dysfunction, ejaculatory dysfunction, or decreased libido. However, breast enlargement caused the patient to become withdrawn and to avoid outdoor activities and social interactions. After diagnosis, a reduction mammaplasty was performed, which decreased the size of the breasts and alleviated the patient's psychological distress. We also found that the majority of PAIS patients had poor height development, with the majority of them under 170 cm tall. Saha et al. (11) also reported a case of PAIS, a 22-year-old with a height of 162 cm, gender is female (A karyotype revealed 46 XY). Unlike the cases we reported:excessive hair growth on the face and chest,the patient had been shaving regularly once every two weeks for the last 6-7 years (Ferriman-Gallwey score:14). Breast development was Tanner stage 3 and axillary hair were Tanner stage 4. The pelvic examination results were similar to those of the cases we reported. Due to the inhibitory actions of AMH in utero (7th week) (produced by Sertoli cells of the testes), there is an absence of the proximal vagina, cervix, uterus, and fallopian tubes too. The lower blind-ending vagina, which is present in such patients, is derived from the urogenital sinus. Therefore, further studies are warranted to determine whether this is related to endocrine or hormone abnormalities. Due to the ambiguity in gender identity, adult PAIS patients experience a greater sense of social inferiority than CAIS patients (12).

AIS is diagnosed by a 46,XY karyotype, normal or increased serum testosterone levels, and mildly elevated LH levels (13). Additionally, due to the excessive production of testosterone and the peripheral aromatization of estrogen, mildly elevated E2 and normal or mildly elevated FSH levels may also be observed. In terms of differential diagnosis, Kennedy disease (KD), also known as spinal bulbar muscular atrophy (SBMA), is a rare X-linked recessive neurodegenerative disease characterized by pathological neuromuscular changes that affect men. KD patients with PAIS are extremely rare, and genetic testing is the gold standard for diagnosing KD (14). Furthermore, 5α-reductase deficiency is also a rare type of DSD with a 46,XY karyotype. However, individuals with this condition gradually develop male sexual characteristics after puberty due to testosterone, resulting in progressive masculinization, voice changes, increased muscle mass, enlarged phallus, and sparse or absent pubic, axillary, and facial hair, along with possible fertility issues (15). Case 16 was an extremely rare case of Klinefelter syndrome (KS) with PAIS. KS, also known as congenital seminiferous tubule dysgenesis or congenital testicular dysgenesis syndrome, is the most common sex chromosome abnormality in men. The most common karyotype is 47,XXY, which was first described by Klinefelter in 1942 (16). For individuals with a 47,XXY karyotype, variations in the AR gene can also lead to varying degrees of aberrant sex development (17). However, cases of KS with PAIS are extremely rare both in China and abroad (18).

Ahsan et al. (19) recently reported a case involving a non-consanguineous family with six “sisters”, with two of the youngest sisters presenting with prominent jawlines, laryngeal prominences, deep voices, and Tanner stage II clitoromegaly. Both sisters had a 46,XY karyotype. In addition, four of the children presented with primary ovarian insufficiency (POI), and two of them showed significantly elevated testosterone levels. Ultrasound and MRI scans revealed the absence of a uterus and ovaries, in addition to the presence of prostates and testes located bilaterally in the inguinal region. The simultaneous occurrence of POI and AIS within a single family has not been reported before. Therefore, the authors also underscored the importance of carefully evaluating all family members presenting with similar symptoms to avoid missed diagnoses.

Patients with PAIS typically have external genitalia characteristic of females. Orchiectomy before puberty prevents clitoromegaly during puberty and reduces the emotional discomfort associated with clitoromegaly during adolescence (3). Estrogen replacement therapy is required for these patients, and the appropriate timing for hormone replacement therapy should be promptly determined after gender assignment under the guidance of a pediatric endocrinologist. All patients with a 46, XY karyotype and female gender assignment need to receive hormone replacement therapy. Our treatment process, apart from lacking specific psychiatric counselling, was basically consistent with what was reported by Saha et al. (11), mainly consists of gonadectomy, vaginal dilatation/plasty, oestrogen therapy. Furthermore, such patients will not be able to get pregnant in the future, and infertility is the main problem. Comprehensive psychiatric assessment and counselling can significantly help alleviate distress. In China, once a child's sex is confirmed after birth, it will accompany them throughout their life. Regardless of the reason for their gender change, the child may become distant from their classmates, colleagues, or friends and may even face ridicule. The surgical procedure for such patients to become male is highly complex, whereas the female conversion procedure is generally considered relatively straightforward according to academic consensus. Therefore, the surgical treatment for intersex conditions, procedures such as microphallus excision, vulvar reconstruction, and vaginoplasty are typically performed. In this study, 7 of the 17 patients received treatment to align their social identity with female characteristics. Although a small phallus may be visible at the clitoral site, the sensory nerves attached to the medial side of the clitoral skin should be preserved as much as possible during vulvoplasty. Proper separation of blood vessels, excision of the enlarged clitoral corpus cavernosum, and suturing and fixing the clitoral glans to the pubic symphysis are key to improving the sensitivity of the preserved clitoris and the patient's quality of life after surgery. By performing needle prick and sensory evoked potentials tests, Altwein et al. (20) confirmed that the clitoris exhibited favorable responses to stimulation after surgery. Due to technical reasons, the patient in this case underwent excision of the microphallus near its base, and the surgical method proposed by Altwein et al. was not used. In the past, the vaginal reconstruction approach in plastic surgery has involved using a penis-scrotum flap, which makes full use of the patient's own external genital tissues. The reconstructed vagina and female external genitalia have a good shape and function; this is also a relatively ideal surgical method. However, the patient's microphallus in this case was only 3.0 cm long, the testes were located in the inguinal canal, and there was no scrotum in the external genital area; therefore, this surgical method is relatively difficult to apply. In addition, phallic size reduction is a standard surgical procedure that can preserve the neurovascular bundle (NVB) in the microphallus, thus having a minimal impact on the sensitivity of the later-formed clitoris. This surgery is also commonly applied in cases of congenital adrenal hyperplasia (CAH). Therefore, the patient reported in this article should not have the microphallus completely removed. Early diagnosis of AIS and appropriate selection of personalized treatment are vital for these patients' prognoses.

## Data Availability

The raw data supporting the conclusions of this article will be made available by the authors, without undue reservation.
